# Morphology, mating system and taxonomy of *Volvox africanus* (Volvocaceae, Chlorophyceae) from Thailand

**DOI:** 10.1186/s40529-022-00332-1

**Published:** 2022-01-21

**Authors:** Hisayoshi Nozaki, Wuttipong Mahakham, Wirawan Heman, Ryo Matsuzaki, Masanobu Kawachi

**Affiliations:** 1 Department of Biological Sciences, Graduate School of Science, The University of Tokyo, Bunkyo-ku, Tokyo, Japan; 2 Biodiversity Division, National Institute for Environmental Studies, Tsukuba, Ibaraki Japan; 3 Department of Biology & Applied Taxonomic Research Center, Faculty of Science, Khon Kaen University, Khon Kaen, Thailand; 4 Department of Science and Mathematics, Faculty of Science and Health Technology, Kalasin University, Mueang Kalasin, Thailand; 5 Faculty of Life and Environmental Sciences, University of Tsukuba, Tsukuba, Ibaraki Japan

## Abstract

**Background:**

The oogamous green algal genus *Volvox* exhibits extensive diversity in mating systems, including heterothallism and homothallism with unisexual (male and/or female) and/or bisexual spheroids. Although four mating systems have been recognized worldwide in strains identified as “*Volvox africanus*”, most of these strains are extinct. However, we previously rediscovered two types of the four mating systems (heterothallic, and homothallic with male and bisexual spheroids within a clone) from an ancient Japanese lake, Lake Biwa.

**Results:**

Here, we obtained strains exhibiting the third mating system (homothallic with unisexual male and female spheroids within a clone) from a freshwater area of Kalasin Province, Thailand. When sexual reproduction was induced in the present Thai strains, both male and female unisexual spheroids developed to form smooth-walled zygotes within a clonal culture. Phylogenetic analyses of the internal transcribed spacer region-2 of nuclear ribosomal DNA sequences from all four mating systems, including the extinct strains, resolved the third mating system is basal or paraphyletic within the homothallic clade.

**Conclusions:**

The present morphological and molecular data of the Thai strains indicate that they belong to the homothallic species *V. africanus*. The phylogenetic results suggested that third mating system (homothallic with separate male and female sexual spheroids) may represent an initial evolutionary stage of transition from heterothallism to homothallism within *Volvox africanus*. Further field collections in geologically stable intracontinental regions may be fruitful for studying diversity and taxonomy of the freshwater green algal genus *Volvox*.

## Background

Evolutionary transitions in the mating systems of a wide range of eukaryotic taxa are a crucial topic in the biological sciences (Wittenberger 1979; Barrett 2010). Freshwater algae are convenient research materials for examining sexual habit, as sexual reproduction can be easily induced under controlled laboratory conditions (Stein 1973). Thus, classical genetic studies of algae have primarily used the unicellular green algal genus *Chlamydomonas* (Harris 1989), and subsequent extensive molecular genetic studies of *C. reinhardtii* resolved its mating-type locus and mating-type specific genes (Ferris and Goodenough 1994, 1997). *Chlamydomonas* species are isogamous without differentiation between males and females, but the closely related multicellular volvocine lineage includes anisogamous genera and the oogamous genus *Volvox* that are diverse in their mating systems (Harris 1989; Hanschen et al. 2018).

Starr (1971) reported four mating systems in worldwide strains of a volvocine alga identified as the single species “*Volvox africanus*” (“VxAf”). Although Coleman (1999) analyzed the internal transcribed spacer region (ITS)-2 of nuclear ribosomal DNA (rDNA) sequences from the four mating systems of “VxAf”, most of the “VxAf” strains deposited in UTEX (Starr and Zeikus 1993) were unavailable (Nozaki et al. 2015a). This problem was partially resolved when new “VxAf” strains representing the two mating systems were established from field-collected water samples in an ancient Japanese lake, Lake Biwa (Nozaki et al. 2015a). One of the two types is homothallic with male and bisexual spheroids and was identified as *Volvox africanus* G.S. West, whereas the other has been described as the new heterothallic species *Volvox reticuliferus* Nozaki (Nozaki et al. 2015a) (Table 1). However, details of morphology and sexual reproduction of “VxAf” strains with the other two mating systems (homothallic with separate male and female sexual spheroids, and homothallic with only bisexual spheroids) have remained unresolved.

**Table 1:** Four types of mating systems and their taxonomy in “VxAf” (*Volvox africanus* and *V. reticuliferus*)

Mating system (Abbreviation)	Heterothallic mating system (Hetero)	Homothallic mating system I (Homo I)	Homothallic mating system II (Homo II)	Homothallic mating system III (Homo III)
Sexual spheroids produced	Male and female spheroids by different genotypes	Male and bisexual spheroids by a single genotype	Male and female spheroids by a single genotype	Bisexual spheroids
Individual sheaths of the spheroid matrix	Confluent or indistinct	Distinct	Distinct	–
Zygote walls	Reticulate	Smooth	Smooth	–
*Volvox* species classified	*V. reticuliferus*	*V. africanus*	*V. africanus*	*V. africanus*
Strains [origins] analyzed in this study (Figs. 3, 4)	NIES-3781, NIES-3782, NIES-3783 [Lake Biwa, Japan]; UTEX 1890–1891 [Small pond, Australia]	NIES-3780 [Lake Biwa, Japan], UTEX 1893 [Dry pond, India]	1101-NK-1 (= NIES-4467), 1101-NZ-11 (= NIES-4468) [Marsh, Thailand]; UTEX 1889 [Shallow pond, USA]	UTEX 1892 [Ecca Pass, South Africa]
References	Starr (1971), Nozaki et al. (2015a)	Starr (1971), Nozaki et al. (2015a)	Starr (1971), the present study	Starr (1971)

UTEX 1893, 1889 and 1892 have been deceased and lack information of individual sheaths and zygote morphology (Nozaki et al. 2015a)

Very recently, we obtained “VxAf” strains of the third mating system (homothallic with separate male and female sexual spheroids; Table 1) from a freshwater habitat in Kalasin Province, in the interior continental region of Thailand. Our morphological and phylogenetic data identified these Thai strains as *V. africanus,* and this type of homothallic mating system may be ancestral within *V. africanus*. The morphology, mating system, and taxonomy of these homothallic strains of *V. africanus* are described in this report.

## Methods

### Establishment of cultures and morphological observations

Water samples (pH 6.85; temperature 30.5 °C) were collected from a marsh in Nong Ya Ma, Yang Talat District, Kalasin Province, Thailand (16° 28ʹ 14.55ʺ N, 103° 16ʹ 25.55ʺ E) on 1 November 2019. Clonal cultures of *V. africanus* (strains 1101-NK-1 and 1101-NZ-11) were established from the water sample using the pipette washing method (Pringsheim 1946). The strains are available as NIES-4467 (1101-NK-1) and NIES-4468 (1101-NZ-11) in the Microbial Culture Collection of the National Institute for Environmental Studies, Japan (Kawachi et al. 2013). Cultures were grown in 18 × 150-mm screw-cap tubes containing 10–11 mL artificial freshwater-6 (AF-6) or Volvox thiamin acetate (VTAC) medium (Kawachi et al. 2013), at 25 °C under a 14:10-h light:dark schedule, under cool-white fluorescent lamps (with a color temperature of 5000 K) at an intensity of 80–130 μmol m^−2^ s^−1^. The cultures were initially maintained in AF-6 medium. For morphological observations, possible contaminant bacteria were removed from the cultures by squeezing a young, unhatched spheroid out of the parental spheroid and washing the spheroid using the pipette washing method. The young spheroid was then grown in 10–11 mL VTAC medium. Asexual spheroids were observed in actively growing cultures in VTAC medium, as described previously (Nozaki et al. 2015a). To induce production of sexual spheroids, 0.3–0.8 mL actively growing culture in VTAC medium (the volume depended on the number and density of spheroids in the culture) at 25 °C were inoculated with 10–11 mL urea soil Volvox thiamine medium (Nozaki et al. 2015b). This culture was then grown at 20 °C under a 14:10-h light:dark schedule under cool-white fluorescent lamps at an intensity of 50–70 μmol m^−2^ s^−1^. Sexual spheroids usually developed within 20 days. Light microscopy was conducted using the BX60 microscope (Olympus, Tokyo, Japan) equipped with Nomarski interference optics. To examine the form of individual sheaths in the gelatinous matrix in spheroids, approximately 20 μL cultured material was mixed with 4–10 μL 0.002% (w/v in distilled water) methylene blue (1B-429 Waldeck GmbH & Co Division Chroma, Münster, Germany). Spheroid cells were counted as described previously (Smith 1944; Nozaki 1988; Nozaki et al. 2019).

For transmission electron microscopy, an actively growing cultured sample was pre-fixed with 1% glutaraldehyde and post-fixed with 2% OsO_4_ and examined as in the previous study (Nozaki et al. 2015a) except for using 4% samarium chloride solution in distilled water instead of saturated solution of uranyl acetate (Matsuzaki et al. 2014).

### Molecular experiments

To infer the phylogenetic position and origin of the Thai strains of *V. africanus*, we analyzed ITS-2 and 6021 base pairs of the coding regions of five chloroplast genes (large subunit of RuBisCO, adenosine triphosphate synthase beta subunit, photosystem I P700 chlorophyll a apoprotein A1, photosystem I P700 chlorophyll a apoprotein A2, and photosystem II CP43 reaction center protein genes) as in previous studies (Nozaki et al. 2015a; Yamamoto et al. 2021), except that the alignments included an additional operational taxonomic unit of the Thai strains of *V. africanus*. New sequences from the Thai strains were determined based on direct sequencing of polymerase chain reaction products, as described previously (Nozaki et al. 2015a; Yamamoto et al. 2021), and are available under Accession numbers LC631309-LC631310 (ITS rDNA for 1101-NZ-11 and 1101-NK-1) and LC631311-LC631315 (five chloroplast genes for 1101-NZ-11). The alignments of ITS-2 and the five chloroplast genes are available from TreeBASE (https://treebase.org/treebase-web/home.html; study ID: 28191). Designation of the outgroup was performed as described previously (Nozaki et al. 2015a; Yamamoto et al. 2021). Maximum-likelihood analyses based on the ITS rDNA and chloroplast multigene alignments were performed using MEGA X (Kumar et al. 2018), with 1000 replicates of bootstrap analyses (Felsenstein 1985). In addition, Bayesian inference of ITS-2 was conducted using MrBayes 3.2.7a (Ronquist et al. 2012), as described in a previous study (Nozaki et al. 2015a). Timetree analysis was performed using the chloroplast multigene data set by MEGA X, as in a previous study (Yamamoto et al. 2021). The secondary structures of ITS-2 were predicted as described previously (Nozaki et al. 2015a).

## Results

### Asexual spheroids

**Fig. 1:**
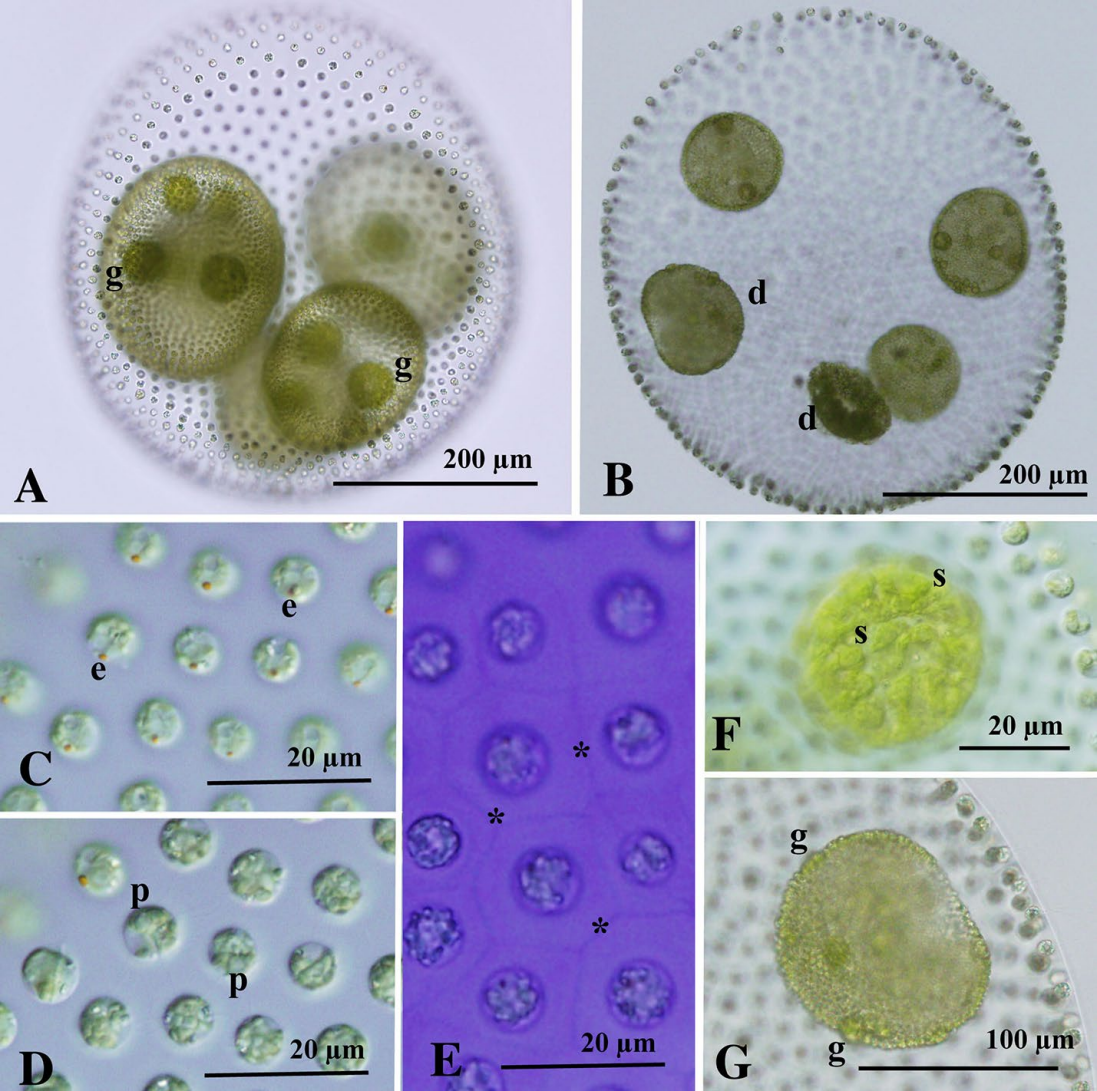
Asexual spheroids of *Volvox africanus* G.S. West strains 1101-NK-1 (**A**) and 1101-NZ-11 (**B**–**G**) from Thailand. **A**, **B**, **E**, **G** Bright-field microscopy. **C**, **D**, **F** Nomarski differential interference contrast microscopy. **A** Parental spheroid containing daughter spheroids with gonidia (g). **B** Parental spheroid with developing embryos (d). **C**–**E** Part of spheroids. **C** Surface view of somatic cells showing an eyespot (e). **D** Optical section of somatic cells showing a pyrenoid (p) in the chloroplast. **E** Front view of somatic cells with individual sheaths (asterisks). Stained with methylene blue. **F** Surface view of gonidium showing radial striations (s). **G** Pre-inversion embryo or plakea. Note that differentiation of gonidia (g) of the next generation is evident

The Thai strains of *V. africanus* produced asexual spheroids that were ovoid, subspherical, or ellipsoidal in shape (Fig. 1A, B). The spheroid was up to 550 μm in length and consisted of approximately 1500–6000 somatic cells and 2–8 reproductive cells (gonidia). Gonidia were generally arranged in one or two tiers. When arranged in two tiers, one was positioned primarily at the equator of the spheroids and the other within the posterior half of the spheroid (Fig. 1A, B); the gonidia of the posterior tier often showed delayed embryogenesis. The cells lacked cytoplasmic bridges between them and were embedded in individual sheaths at the periphery of the gelatinous matrix (Fig. 1C, D). Individual sheaths were rectangular or hexagonal in shape and compactly arranged without fenestrations between them in the frontal view (Fig. 1E). Somatic cells had two equal flagella and a cup-shaped chloroplast with a single basal pyrenoid and a single eyespot, and they measured up to 8 μm in length (Fig. 1C, D). Gonidia had radial striations on the surface of the chloroplast in the frontal view (Fig. 1E). During the plakeal stages of the developing embryo, gonidia of the next generation were evident (Fig. 1G).

### Sexual spheroids

**Fig. 2:**
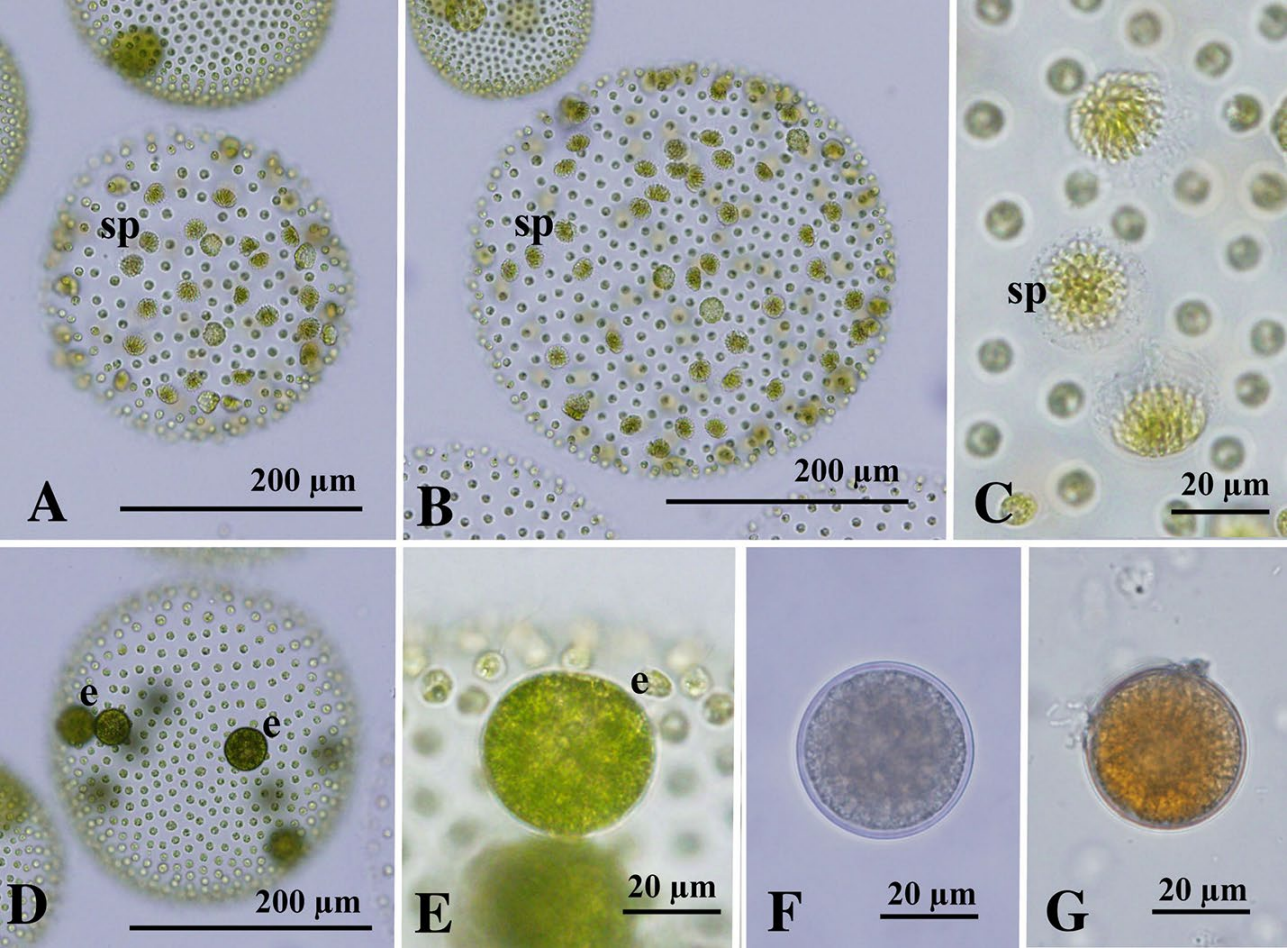
Sexual spheroids of *Volvox africanus* G.S. West strains 1101-NK-1 (**A**–**F**) and 1101-NK-1 x 1101-NZ-11 (**G**) from Thailand. **A**–**G** Bright-field microscopy. **A**–**C** Male spheroids with sperm packets (sp). **D**, **E** Female spheroids with eggs (e). **F**, **G** Mature zygotes with a smooth cell wall

Both male and female sexual spheroids were unisexual and developed within a single clonal culture. Male spheroids were generally 500–1300-celled and subspherical or ovoid in shape, with 60–100 sperm packets distributed almost throughout the surface of the spheroid (Fig. 2A, B). Sperm packets were hemispherical in shape and were composed of approximately 100–200 spindle-shaped male gametes (sperm) (Fig. 2C). Female spheroids contained 500–1500 cells and usually 7–11 eggs within the posterior three-fifths (Fig. 2D, E). Production of zygotes (thick-walled cells) were rare under the present culture conditions, but they were found even within the single clonal culture. Zygotes measured approximately 30 μm in diameter and had a smooth cell wall (Fig. 2F, G).

### Transmission electron microscopy

**Fig. 3:**
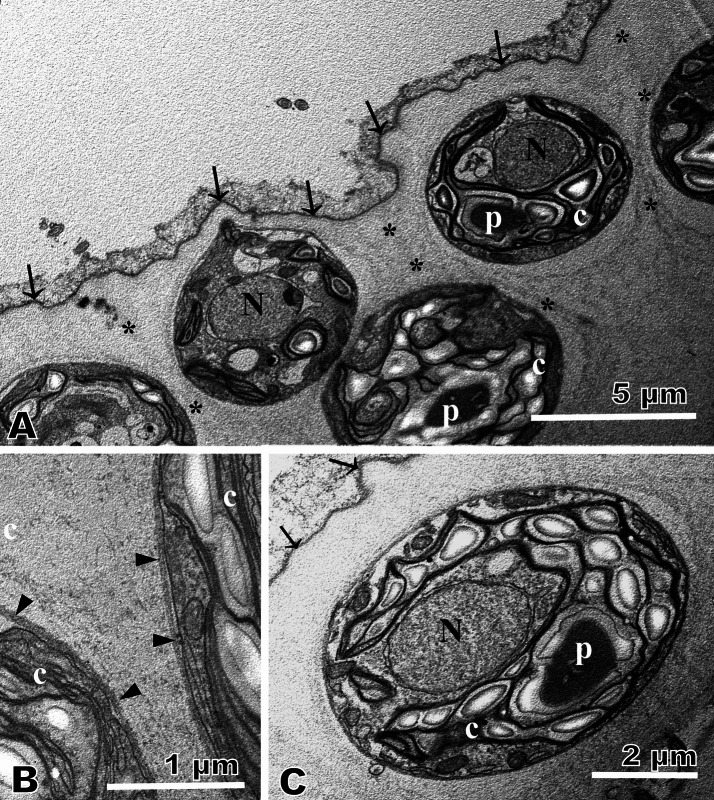
Transmission electron microscopy of asexual spheroids of *Volvox africanus* G.S. West strain 1101-NZ-11 from Thailand. **A**–**C** Whole spheroid is surrounded by a tripartite layer (colonial boundary) of the extracellular matrix (arrows). Each protoplast is enclosed tightly by a thin layer (cellular envelope) of the matrix (arrowheads) and has a nucleus (N) and a chloroplast (c) with a pyrenoid (p). Note that the matrix between cells forms individual sheath (asterisks). **A** Section of peripheral region showing cells and extracellular matrix. **B** Details of cellular envelopes. **C** Longitudinal section of a cell

The entire spheroid of the present Thai strain was surrounded by a tripartite boundary (colonial boundary) of the extracellular matrix as (Fig. 3A) as in other volvocacean algae (Nozaki and Kuroiwa 1992). Just beneath the colonial boundary, cells were observed with profiles of a chloroplast, mitochondria, and a nucleus inside the cell membrane (Fig. 3A–C). Each cell was tightly enclosed by a thin layer [cellular envelope (Nozaki and Kuroiwa 1992)] of the matrix (Fig. 3B). At the peripheral region of the spheroid, the matrix within the space between the cells formed a fibrillar layer (Fig. 3A) as in *V. africanus* from Lake Biwa (Nozaki et al. 2015a), representing the individual sheath observed under the light microscopy (Fig. 1E).

### Phylogenetic analyses

**Fig. 4:**
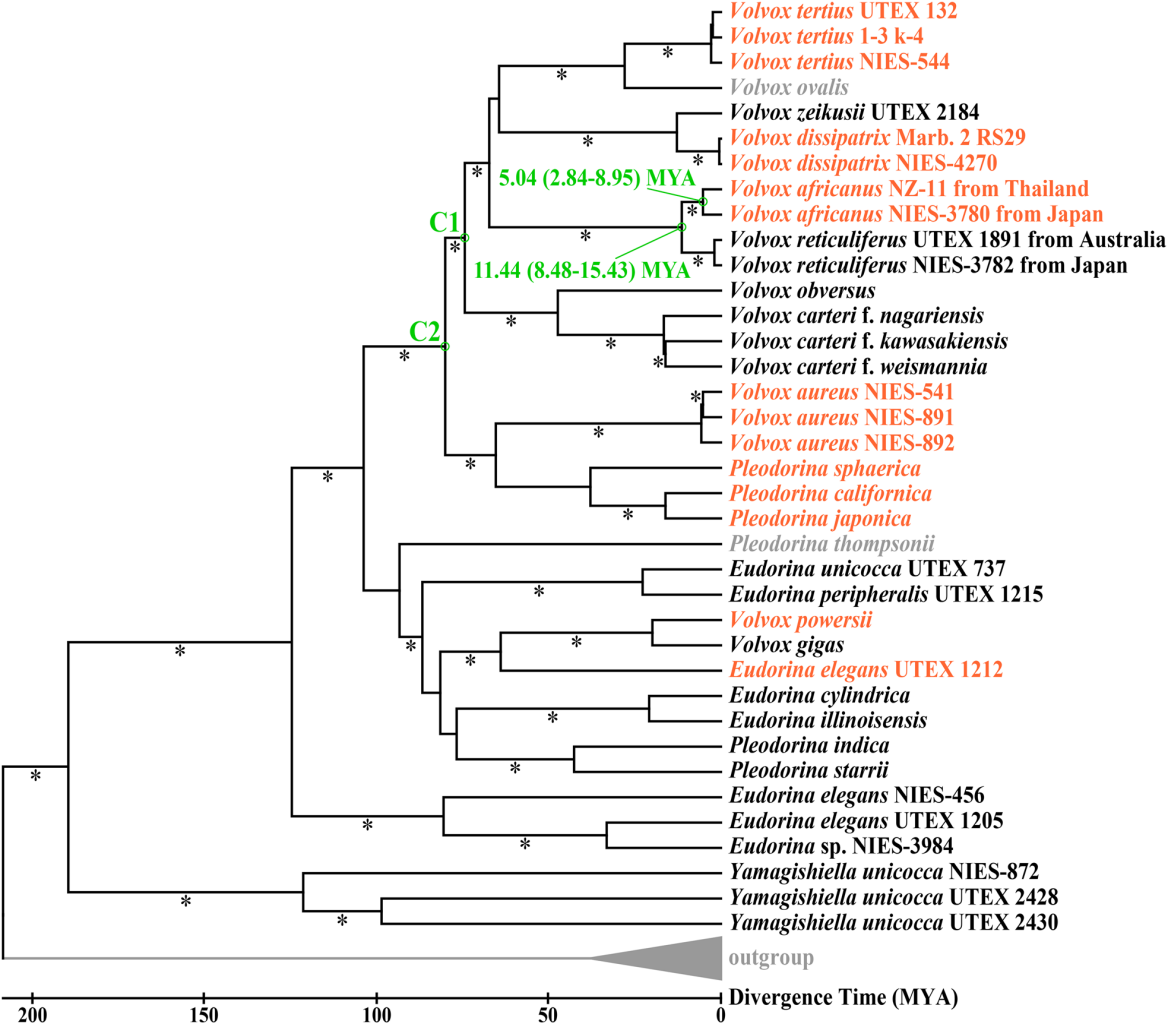
Timetree analysis of advanced members of the colonial volvocine algae including the Thai strains of *Volvox africanus*. Note that the divergence time between heterothallic *Volvox reticuliferu*s and homothallic *V. africanus* and the divergence between Thai and Japanese strains of *V. africanus* are “11.44 (8.48–15.43) MYA” and “5.04 (2.84–8.95) MYA”, respectively. Species names in orange or black indicate homothallic or heterothallic sexuality, respectively. Tree topology was inferred by maximum likelihood (ML) analyses of 6021 base pairs of five chloroplast genes from 47 operational taxonomic units [TreeBASE (Vos et al. 2010, 2012) ID 28191] with a model selected by MEGA X (Kumar et al. 2018). Asterisks on branches indicate 80% or more bootstrap values (based on 1000 replicates) by the ML analyses. A timetree was inferred by applying the RelTime method (Tamura et al. 2012, 2018) to the ML phylogenetic tree whose branch lengths were calculated using the ML method and the General Time Reversible substitution model (Nei and Kumar 2000). The timetree was computed using two calibration constraints [C1 (65–90 MYA) and C2 (50–90 MYA)] based on TimeTree: the Time Scale of Life <http://www.timetree.org/> (Liss et al. 1997; Herron et al. 2009). Outgroup was designated as in the previous study (Yamamoto et al. 2021). Evolutionary analyses were conducted in MEGA X (Kumar et al. 2018)

Based on the chloroplast multigene data set, phylogenetic relationships within the anisogamous/oogamous members of the colonial volvocine algae [*Eudorina* group (Nozaki et al. 2015a)] were resolved as described in a previous study (Yamamoto et al. 2021), except for the addition of the Thai strain of *V. africanus*. Four different isolates of “VxAf” formed a robust monophyletic group (“VxAf” lineage) within *Volvox* sect. *Merrillosphaera* (Fig. 4). The “VxAf” lineage was subdivided into two sister groups: one homothallic *V. africanus* composed of the Japanese and Thai strains and the other two strains of heterothallic *V. reticuliferus*. Our timetree analysis dated the origin of “VxAf” and the separation between Thai and Japanese strains of *V. africanus* as approximately 11 MYA and 5 MYA, respectively (Fig. 4).

**Fig. 5:**
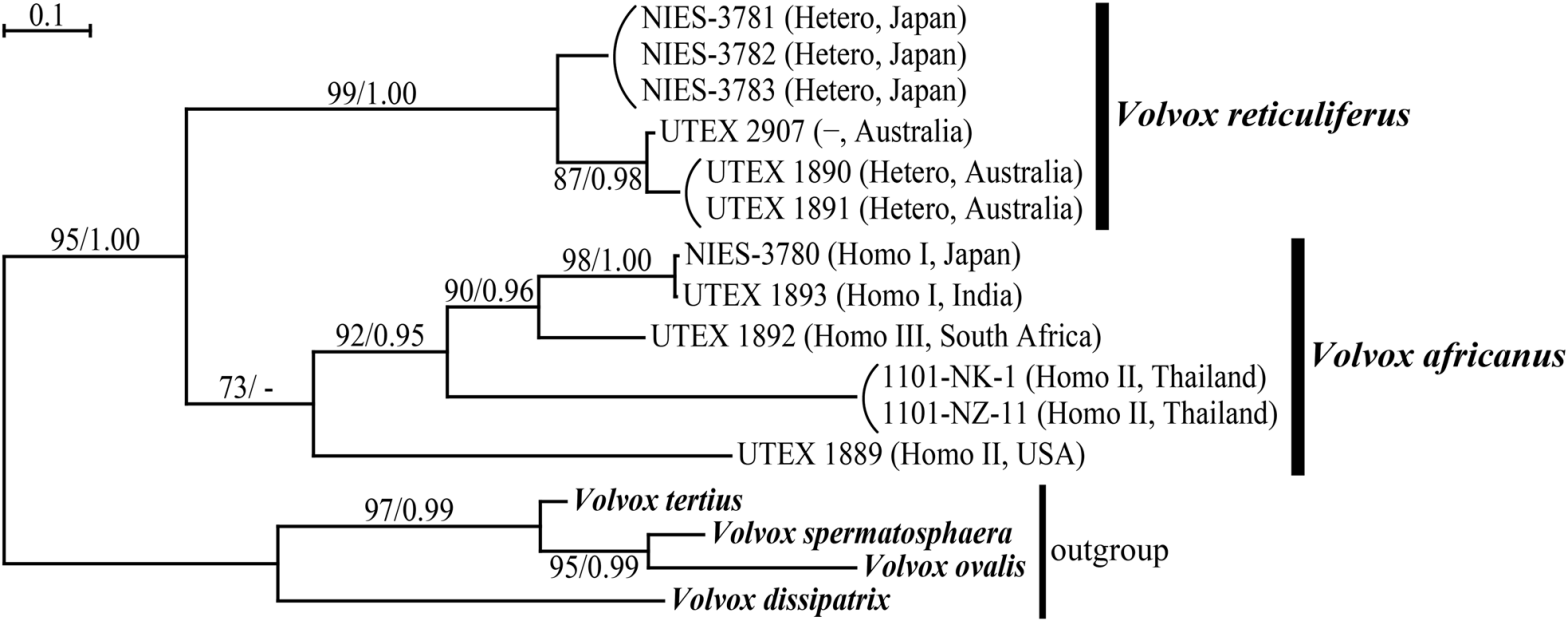
Phylogenetic relationships of four types of mating systems (Table 1) of *Volvox africanus* (homothallic) and *V*. *reticuliferus* (heterothallic), as inferred from internal transcribed spacer region 2 of nuclear ribosomal DNA. UTEX 2907 originating from Australia (https://utex.org/) lacks information of sexual reproduction (Nozaki et al. 2015a), but it was classified as *V. reticuliferus* based on vegetative morphology and phylogeny (Nozaki et al. 2015a). The tree was constructed by ML method (based on T92 + G model). The alignment is available in TreeBASE (Vos et al. 2010, 2012; ID 28191). Branch lengths are proportional to the genetic distances, which are indicated by the scale bar above the tree. Numbers on the left or right side at the branches represent bootstrap values (≥ 50%, based on 1000 replicates) obtained with the ML analysis or posterior probabilities (≥ 0.95) of Bayesian inference, respectively

The ITS-2 phylogenetic analysis included five “VxAf” strains of UTEX [Culture Collection of Algae at the University of Texas at Austin (Starr and Zeikus 1993)], in which Starr (1971) observed four mating systems (Fig. 5). As in the chloroplast multigene phylogeny, two sister clades were resolved in the “VxAf” lineage: homothallic *V. africanus* and heterothallic *V. reticuliferus* (Fig. 5). However, the most basal position of UTEX 1889 within the homothallic clade was moderately resolved (with only a 73% bootstrap value using the maximum-likelihood method) (Fig. 5). The strains of two homothallic mating systems with production of bisexual spheroids (Homo I and III, with and without male unisexual spheroids, respectively) constituted a derived lineage, to which two non-sister strains [Thai and Missouri (UTEX 1889; Starr 1971) strains] of the homothallic mating system with male and female unisexual spheroids were basal (Fig. 5).

### Compensatory base changes (CBCs) in nuclear rDNA ITS-2

**Fig. 6:**
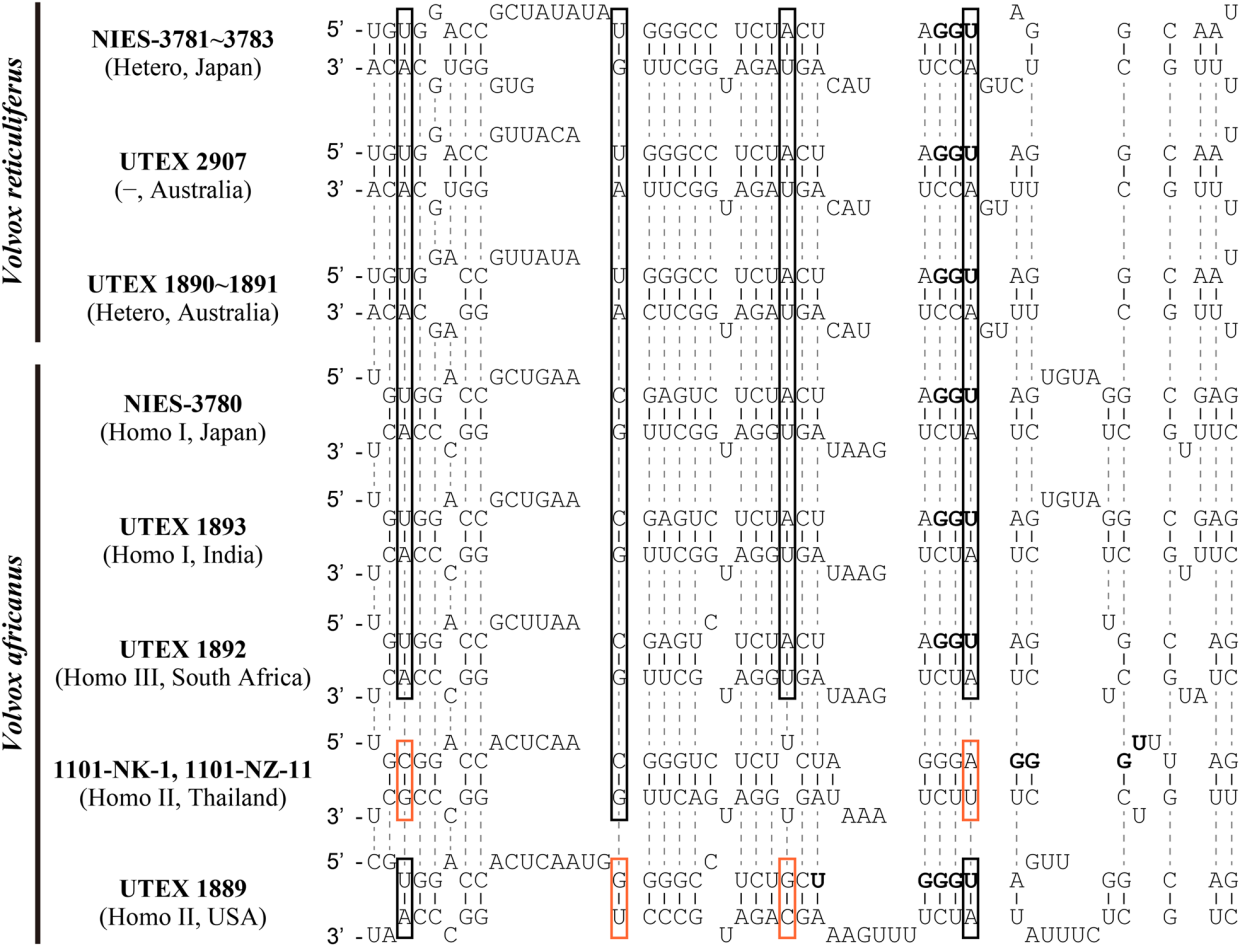
Comparison of the most conserved region [near the YGGY motif of helix III (Coleman 2009)] of nuclear rDNA ITS-2 secondary structure between strains of four mating systems of *Volvox africanus* and *V. reticuliferus* (Table 1). Note the modified YGGY motif (boldface). For secondary structures of ITS-2, see Nozaki et al. (2015a). Red and black open boxes indicate compensatory base changes between Homo II strains (from Thailand and USA) and those of other sexual systems

As resolved previously (Nozaki et al. 2015a), CBCs were not detected in the most highly conserved region of nuclear rDNA ITS-2 [helix III (Nozaki et al. 2015a)] among operational taxonomic units within the heterothallic clade identified as *V. reticuliferus* (Fig. 6). However, the Thai strains or UTEX 1889 had two or three CBCs compared with other strains within the homothallic clade identified as *V. africanus* (see below) (Fig. 6).

## Discussion

### Taxonomy

*Volvox reticuliferus* is clearly distinguished from *V. africanus*, based on its morphological traits (reticulate zygote walls and confluent or indistinct individual sheaths), heterothallic mating system and phylogeny (Table 1) (Nozaki et al. 2015a). Even though information regarding zygote morphology and/or mating systems is lacking for UTEX 1890, 1891, and 2907 strains, they were identified as *V. reticuliferus* based on vegetative morphology (individual sheaths) and phylogeny (Nozaki et al. 2015a). In the homothallic clade, however, such vegetative morphology has previously been obtained in only one (Homo I) of the three homothallic mating systems of “VxAf” by Starr (1971), due to the extinction of strains belonging to other homothallic mating systems (Homo II and III) (Nozaki et al. 2015a). The present study established new strains of “VxAf” originating from Thailand and revealed their homothallic sexual habit (Homo II) and morphological characteristics (smooth zygote walls and compact and angular individual sheaths; Figs. 1, 2 and 3) that are consistent with those of *V. africanus* (Table 1) (Nozaki et al. 2015a). In addition, the divergence date between mating systems Homo I (Lake Biwa, Japan) and Homo II (Kalasin, Thailand) is younger than those between sister species within the anisogamous/oogamous members of the multicellular volvocine algae (Fig. 4). Thus, “VxAf” strains of all homothallic mating systems (Homo I–III; Table 1) should be classified as *V. africanus* based on their monophyletic status (Figs. 4, 5) and common morphological/phenotypical characteristics (Table 1), even though their mating systems vary in the production of sexual spheroids (Table 1), and their ITS-2 rDNA demonstrated the presence of CBCs between them (Fig. 6). This taxonomic conclusion is consistent with the monophyletic morphological species concept in microalgae (Nozaki et al. 1998).

### Evolution of homothallic mating systems in *Volvox*

Volvocine algae, especially *Volvox* species are an excellent model for studying the evolution of sexual dimorphism and mating systems. Some species of *Volvox* are heterothallic species, in which different genotypes produce either eggs or sperm, while others are homothallic species with a single genotype producing both eggs and sperm (Hanschen et al. 2018). The “VxAf” lineage is an excellent model for investigating the evolutionary transition between heterothallism to homothallism as it represents both homothallism and heterothallism within a closely related group (Figs. 4, 5).

Based on the genome comparison of sex-determining regions in male and female determining chromosomes of heterothallic *V. reticuliferus* and the sex-determining region-like region of homothallic *V. africanus*, the evolutionary transition from heterothallism to homothallism in the “VxAf” lineage was unambiguously resolved (Yamamoto et al. 2021). In addition, the present phylogenetic analyses of ITS-2 demonstrated that two strains of the homothallic mating system with separate or unisexual male and female sexual spheroids (Homo II) are basal and paraphyletic and may represent the ancestral state of the homothallism in the “VxAf” lineage (Fig. 5). The heterothallic mating system of “VxAf” (*V. reticuliferus*) also produces unisexual male and female sexual spheroids as in Homo II (Table 1). Thus, during the initial stage of the evolutionary transition from heterothallism to homothallism in the “VxAf” lineage, the ancestor of the “VxAf” lineage may have first evolved this type of homothallic mating system (Homo II) from the heterothallic mating system (Hetero) without modification of the form of sexual spheroids (unisexual male and female). Bisexual spheroids may have subsequently evolved in the homothallic ancestor of the “VxAf” lineage.

## Conclusions

Algal diversity in Kalasin Province shows that freshwater bodies of this region contain a variety of volvocine and other interesting green algae (Heman 2015). The present re-discovery of the homothallic “VxAf” strains with unisexual male and female spheroids is based on field collections in a freshwater habitat of an intracontinental region of Thailand. We also found a new species of *Volvox* sect. *Volvox*, *V. longispiniferus* Nozaki & Mahakham, from the same field collection (Nozaki et al. 2020). In addition, two species of “VxAf” were collected in an ancient lake, Lake Biwa, Japan (Nozaki et al. 2015a). Thus, freshwater habitats in such geologically stable intracontinental regions may be fruitful for *Volvox* diversity.

## Data Availability

New sequence data, alignments used for our phylogenetic analyses, and new strains are available under the DDBJ/EMBL-EBI/NCBI Accession numbers (LC631309–LC631315), TreeBASE study ID (28191), and NIES Collection strain designations (NIES-4467–NIES-4468), respectively. All other relevant data are within the paper.

## Abbreviations

AF-6: Artificial freshwater-6
“VxAf”: “*Volvox africanus*”
CBC: Compensatory base change
ITS: Internal transcribed spacer region
ML: Maximum likelihood
rDNA: Nuclear ribosomal DNA
VTAC: Volvox thiamin acetate

## References

[CR1] Barrett SC (2010). Darwin's legacy: the forms, function and sexual diversity of flowers. Philos Trans R Soc Lond B Biol Sci.

[CR2] Coleman AW (1999). Phylogenetic analysis of "Volvocaceae" for comparative genetic studies. Proc Natl Acad Sci USA.

[CR3] Coleman AW (2009). Is there a molecular key to the level of "biological species" in eukaryotes? A DNA guide. Mol Phylogenet Evol.

[CR4] Felsenstein J (1985). Confidence limits on phylogenies: an approach using the bootstrap. Evolution.

[CR5] Ferris PJ, Goodenough UW (1994). The mating-type locus of *Chlamydomonas reinhardtii* contains highly rearranged DNA sequences. Cell.

[CR6] Ferris PJ, Goodenough UW (1997). Mating type in *Chlamydomonas* is specified by *mid*, the minus-dominance gene. Genetics.

[CR7] Hanschen ER, Herron MD, Wiens JJ, Nozaki H, Michod RE (2018). Repeated evolution and reversibility of self-fertilization in the volvocine green algae. Evolution.

[CR8] Harris EH (1989). The *Chlamydomonas* sourcebook. A comprehensive guide to biology and laboratory use.

[CR9] Heman W (2015) Diversity and distribution of algae in Kalasin province, Thailand. A report submitted to The National Research University & Higher Education Research Promotion (NRU & HERP), Office of the Higher Education Commission, Thailand

[CR10] Herron MD, Hackett JD, Aylward FO, Michod RE (2009). Triassic origin and early radiation of multicellular volvocine algae. Proc Natl Acad Sci USA.

[CR11] Kawachi M, Ishimoto M, Mori F, Yumoto K, Sato M, Noël M-H (2013). MCC-NIES. List of strains.

[CR12] Kumar S, Stecher G, Li M, Knyaz C, Tamura K (2018). MEGA X: molecular evolutionary genetics analysis across computing platforms. Mol Biol Evol.

[CR13] Liss M, Kirk DL, Beyser K, Fabry S (1997). Intron sequences provide a tool for high-resolution phylogenetic analysis of volvocine algae. Curr Genet.

[CR14] Matsuzaki R, Hara Y, Nozaki H (2014). A taxonomic study of snow *Chloromonas* species (Volvocales, Chlorophyceae) based on light and electron microscopy and molecular analysis of cultured material. Phycologia.

[CR15] Nei M, Kumar S (2000). Molecular evolution and phylogenetics.

[CR16] Nozaki H (1988). Morphology, sexual reproduction and taxonomy of *Volvox carteri* f. *kawasakiensis* f. nov. (Chlorophyta) from Japan. Phycologia.

[CR17] Nozaki H, Kuroiwa T (1992). Ultrastructure of the extracellular matrix and taxonomy of *Eudorina*, *Pleodorina* and *Yamagishiella* gen. nov. (Volvocaceae, Chlorophyta). Phycologia.

[CR18] Nozaki H, Ohta N, Morita E, Watanabe MM (1998). Toward a natural system of species in *Chlorogonium* (Volvocales, Chlorophyta): a combined analysis of morphological and *rbcL* gene sequence data. J Phycol.

[CR19] Nozaki H, Matsuzaki R, Yamamoto K, Kawachi M, Takahashi F (2015). Delineating a new heterothallic species of *Volvox* (Volvocaceae, Chlorophyceae) using new strains of “*Volvox africanus*”. PLoS ONE.

[CR20] Nozaki H, Ueki N, Misumi O, Yamamoto K, Yamashita S, Herron MD, Rosenzweig F (2015). Morphology and reproduction of *Volvox capensis* (Volvocales, Chlorophyceae) from Montana, USA. Phycologia.

[CR21] Nozaki H, Takusagawa M, Matsuzaki R, Misumi O, Mahakham W, Kawachi M (2019). Morphology, reproduction and taxonomy of *Volvox dissipatrix* (Chlorophyceae) from Thailand, with a description of *Volvox zeikusii sp. nov*. Phycologia.

[CR22] Nozaki H, Mahakham W, Heman W, Matsuzaki R, Kawachi M (2020). A new preferentially outcrossing monoicous species of *Volvox* sect. *Volvox* (Chlorophyta) from Thailand. PLoS ONE.

[CR23] Pringsheim EG (1946). Pure cultures of algae.

[CR24] Ronquist F, Teslenko M, van der Mark P, Ayres DL, Darling A, Höhna S, Larget B, Liu L, Suchard MA, Huelsenbeck JP (2012). MrBayes 3.2: efficient Bayesian phylogenetic inference and model choice across a large model space. Syst Biol.

[CR25] Smith GM (1944). A comparative study of the species of *Volvox*. Trans Am Microsc Soc.

[CR26] Starr RC, Parker BC, Brown Jr RM (1971). Sexual reproduction in *Volvox africanus*. Contribution in phycology.

[CR27] Starr RC, Zeikus JA (1993). UTEX—the culture collection of algae at the University of Texas at Austin. 1993 List of cultures. J Phycol.

[CR28] Stein JR (1973). Handbook of phycological methods. Culture methods and growth measurements.

[CR29] Tamura K, Battistuzzi FU, Billing-Ross P, Murillo O, Filipski A, Kumar S (2012). Estimating divergence times in large molecular phylogenies. Proc Natl Acad Sci USA.

[CR30] Tamura K, Qiqing T, Kumar S (2018). Theoretical foundation of the RelTime method for estimating divergence times from variable evolutionary rates. Mol Biol Evol.

[CR31] Vos R, Lapp H, Piel W, Tamen V (2010). TreeBASE2: rise of the machines. Nat Prec.

[CR32] Vos RA, Balhoff JP, Caravas JA, Holder MT, Lapp H, Maddison WP, Midford PE, Priyam A, Sukumaran J, Xia X, Stoltzfus A (2012). NeXML: rich, extensible, and verifiable representation of comparative data and metadata. Syst Biol.

[CR33] Wittenberger JF, Marler P, Vandenbergh JG (1979). The evolution of mating systems in birds and mammals. Social behavior and communication.

[CR34] Yamamoto K, Hamaji T, Kawai-Toyooka H, Matsuzaki R, Takahashi F, Nishimura Y, Kawachi M, Noguchi H, Minakuchi Y, Umen JG, Toyoda A, Nozaki H (2021). Three new genomes in the algal genus *Volvox* reveal the fate of a haploid sex-determining region after a transition to homothallism. Proc Natl Acad Sci USA.

